# EXPLAIN Fragile-X: an explorative, longitudinal study on the characterization, treatment pathways, and patient-related outcomes of Fragile X Syndrome

**DOI:** 10.1186/1471-244X-13-339

**Published:** 2013-12-19

**Authors:** Frank Haessler, Franziska Gaese, Michael Colla, Michael Huss, Christoph Kretschmar, Marc Brinkman, Heike Schieb, Helmut Peters, Samuel Elstner, David Pittrow

**Affiliations:** 1 Zentrum für Nervenheilkunde, Klinik für Psychiatrie, Neurologie, Psychosomatik und Psychotherapie im Kindes- und Jugendalter, Universitätsmedizin Rostock, Rostock, Germany; 2 Abt. Psychiatrische Therapie für Menschen mit Geistiger Behinderung, Isar-Amper-Klinikum gGmbH, Klinikum München-Ost, Haar, Germany; 3 Experimental and Clinical Research Center, Charité – Campus Berlin Buch & Department of Psychiatry and Psychotherapy, Charité – Campus Mitte, Berlin, Germany; 4 Rheinhessen-Fachklinik Mainz, Kinder- und Jugendpsychiatrie, Mainz, Germany; 5 Städt. Krankenhaus Dresden-Neustadt, Zentrum für Kinder- und Jugendmedizin - Sozialpädiatrisches Zentrum, Dresden, Germany; 6 Medizinische Abteilung, Novartis Pharma GmbH, Nürnberg, Germany; 7 Berliner Behandlungszentrum der Abteilung für Psychiatrie, Psychotherapie und Psychosomatik, Evangelisches Krankenhaus Königin Elisabeth Herzberge gGmbH, Berlin, Germany; 8 Institut für Klinische Pharmakologie, Medizinische Fakultät, Technische Universität Carl Gustav Carus Dresden, Dresden, Germany

**Keywords:** Observational trial, Longitudinal, Patient-related outcomes, Health care, Outcomes, Ambulatory setting, Quality of life, Caregiver burden

## Abstract

**Background:**

Fragile X syndrome (FXS), caused by a mutation of the *FMR1* gene on the X chromosome, is the most common inherited form of intellectual disability and autism spectrum disorders. Comprehensive data are lacking, however, on the characteristics and management patients with FXS in Germany.

**Methods/design:**

EXPLAIN is a prospective, observational, longitudinal registry with a non-probability sampling approach. It collects data on patient characteristics, therapeutic interventions, psychosocial parameters (including those of family members and caregivers), quality of life of caregiver and patient, caregiver burden, and health economic parameters, such as hospitalisation time. It is designed to include data from 300 patients in ambulatory care from about 50 centres that employ psychiatrists, paediatricians, neurologists, and other relevant specialists, in Germany. The study was initiated in March, 2013. Patients will be followed for at least two years.

**Discussion:**

The registry is expected to provide much-needed data on the characteristics and management of patients with FXS in Germany. It will also allow comparisons with other countries, and will enable gap analyses based on current guidelines for management of these patients.

**Trial registration:**

The ClinicalTrials.gov identifier is NCT01711606.

## Background

Fragile X syndrome (FXS) is a trinucleotide repeat disorder caused by a CGG repeat expansion in the *FMR1* gene on the X chromosome (> 200 repeats in the full mutation, compared with 6–54 repeats in the normal population). This expanded repeat region causes the loss of the product fragile X mental retardation protein (FMRP), which regulates neurotransmitter-activated dendritic translation and synaptic plasticity [1,2]. FXS constitutes the most common inherited form of intellectual disability (ID). It is also one of the most well-known genetic mechanisms underlying autism spectrum disorder [3].

Reduced intelligence of varying degrees constitutes a major symptom of FXS, varying from learning difficulties to severe cognitive impairment [4]. Speech, language, and attention deficits, as well as behavioural problems, frequently occur [5-7]. The incidence of other psychopathological syndromes and disorders is also increased: about 25- 50% of males with FXS meet full diagnostic criteria for autistic disorder [8-10]. Roughly 15% of children with FXS suffer from seizures, although these seizures are relatively benign and resolve upon adulthood [11]. Males and females are both affected by FXS, but in females symptoms are often milder, due to the inactivation of one of the two X chromosomes in female cells.

A definitive diagnosis can be made by a simple blood sample test and DNA analysis by Southern blot or PCR [12]. Therapeutic options are very limited [13]. In a US survey, caregivers reported utilisation of medications for attention deficit disorder, anxiety, hyperactivity, mood swings, anger, depression, seizures, self-injury, or sleep disorders [14]. Further, non-drug therapy such as speech-language therapy or occupational therapy is frequently administered in this patient group [15].

A number of agents have been tested for a variety of indications, including FXS, based on their synaptic mechanisms. These include arbaclofen, RO4917523, ganaxolone, acamprosate, and minocycline. In addition, the novel mGluR5 inhibitor AFQ056 (mavoglurant) is currently being investigated in phase III studies in adults and children with FXS [16,17].

To date, data on the prevalence and status of patients with FXS in Germany and other European countries are limited. Conservative estimations assume that some 20,000 people in Germany suffer from FXS, and that many of these cases have not yet been diagnosed.

### Study aims

Considering this lack of data, we initiated a nationwide observational registry to document and comprehensively assess patients with FXS. The registry focuses on somatomedical and neuropsychiatric aspects, cognitive impairment assessment, and quality of life and patient functioning. Further, economic aspects such as use of resources are documented. Drug utilization and patient adherence to therapy are also addressed, for both existing and new interventions.

Specifically, the registry aims to provide information on the following aspects of FXS patients and FXS intervention in Germany:

• Characterisation of the phenotype of patients with FXS

• Description of patient characteristics (demographics, family history, comorbidity, education, employment status, care and treatment conditions, and insurance status)

• Documentation of therapeutic interventions and their outcomes

• Recording and assessment of psychosocial parameters (including family members and/or caregivers)

• Quality of life of the caregiver and, if possible, the patient

• Health economic parameters and use of resources

## Methods

### Design and setting

The present study is a prospective observational study (registry) in Germany involving physicians from about 50 centres (hospitals and physician practices) experienced in the management of FXS. Specialists from other disciplines are also expected to be involved, such as child/adolescent psychiatrists, general psychiatrists, or physicians in social paediatric centres. A non-probability sampling method is used for centres and for patients.

### Patients

Inclusion criteria are a confirmed diagnosis of FXS by genetic testing (> 200 CGG repeats) and written informed consent to participate. No additional genetic testing is required by this study. Patients of all ages and both genders are eligible.

Patient data will be collected at baseline and approximately every 6 months thereafter. Patients will be followed for at least 2 years.

### Parameters

At the inclusion visit, data on the following parameters will be documented:

• Demographic data (birth year, gender, height, and weight)

• Availability of prior genetic testing results (to confirm FXS diagnosis)

• Status/medical history of relatives (FXS status in siblings, parents, or other family members; selected comorbidities in father or mother; educational degree of parents). No information on premutation in parents is collected.

• Insurance status (statutory health insurance, private insurance)

• FXS patient history (date of diagnosis and symptoms at diagnosis as measured by the six-item checklist of Giangreco et al. [18])

• Information on autistic-like behaviour (using the checklist by Giangreco et al. [18] that specifies the characteristics tactile defensiveness, perseverative speech, hand flapping, and poor eye contact)

• Noteworthy life events in the past six months (e.g. change of caregiver, relocation, death of relatives, change of school or workplace)

• Educational status and employment status of the patient

o Education status (in children/adolescents over 16 years of age)

o Work status (in patients aged ≥ 16 years)

o Pre-school care situation (in children under 6 years of age)

o Education status (in children/adolescents aged 6–17 years)

• Comorbid disorders of the patient (seizures, anxiety, depression, attention deficit disorder, other)

• Neurologic/psychiatric status (if available, at the inclusion visit, information from the patient’s charts on tremor, mental retardation, ataxia, and spasticity will be recorded)

• Previous and current management of FXS

o medical therapy (in particular, for epilepsy, anxiety, depression, and/or attention deficit/hyperactivity disorder, with information on treatment period and effectiveness of treatment)

o non-drug therapy such as psychotherapy, ergotherapy, speech-language therapy, physical therapy, occupational therapy, sociotherapy, music therapy, etc. (with information on start date and units per year).

• Symptoms Check List (SCL) [19]

• Economic parameters: contacts by physicians (various specialties), days of hospitalisation since suspected FXS diagnosis, absence from work

• Results of psychometric testing

o IQ testing: Adaptives Intelligenz Diagnostikum (AID) [20], Hamburg-Wechsler-Intelligenztest für Kinder (HAWIK-R/HAWIE-R) [21], Kaufmann Assessment Battery for Children (K-ABC) [22], Snijders Oomen Test [23], or other IQ tests

o Aberrant Behaviour Checklist (ABC Scales) [24]

• Quality of life questionnaires (available as paper forms in the study file)

o EQ-5D (adults) or EQ-5D-Y (children and adolescents) [25], to be filled out by patient with support from caregiver

o Eltern Belastungs Inventar (EBI): German version of the Parenting Stress Index (PSI) questionnaire, if caregiver is a family member [26]

o Nurses’ Observation Scale for In-patient Evaluation (NOSIE), if caregiver is not a family member [27,28]

o Short version of the International Classification of Functioning, Disability, and Health (Mini-ICF) rating for psychiatric disorders [29]

At the follow-up visits (approximately every 6 months), information on the following parameters will be documented, in the same manner as at baseline:

• Noteworthy life events (none, change of caregiver, change of residence, divorce or marital separation of parents, death of relative, birth of sibling, starting school, change of workplace, other).

• Educational status and employment status of the patient

• Current management of FXS (including treatment of symptoms and concomitant diseases such as seizures and depression)

o medical therapy

o non-medical therapy

• Economic parameters

• Results of psychometric testing (as at the baseline visit)

• Quality of life questionnaires (as at the baseline visit)

### Statistical methods

A Statistical Analysis Plan (SAP) was set up prior to initiation of the registry that specified subgroups for analysis (e.g. by age group or autism degree according to physician rating) and statistical methods to be used. The analysis set will consist of all patients with confirmed FXS diagnosis included in the study. The observational study will be analysed descriptively, using established statistical and epidemiological methods. If inferential statistics such as confidence intervals and/or p-values are calculated, the results will be regarded as descriptive only. No adjustments will be made for multiple comparisons.

Continuous variables will be reported as median with interquartiles and other percentiles, and as mean with standard deviation (SD), together with minimum and maximum values. Categorical variables will be reported as absolute values and relative percentages.

For adverse event (AE) and serious adverse event (SAE) incidence, rates based on the number of included patients and incidence density based on the number of events/ patient years will be calculated. These events, as well as serious drug reactions, will be presented overall and by system organ class and preferred term, according to the Medical Dictionary for Regulatory Activities (MedDRA) [30].

Our aim is to include 300 ambulatory patients from about 50 centres in the registry. A minimum sample size has not yet been determined; it will be determined according to feasibility aspects. Interim analyses are planned after inclusion of 50 and 150 patients. Data will be analysed with STATA 12.0 (Stata Corporation, College Station, Texas, USA).

### Ethics and applicable laws

This non-interventional study is being conducted in accordance with the following regulations and guidelines: (1) codex of the FSA (dated 7 May 2008, BAnz. Nr. 68, S. 1636); (2) recommendations of the Federal Institute for Drugs and Medical Products (BfArmM) and the Paul Ehrlich Institute on the planning, conduct, and analysis of post-marketing studies, dated 7 July 2010 [31]; (3) recommendations of the VFA [Association of Research-based Pharmaceutical Companies, dated 31 Jan 2007 and 23 April 2007) [32]. The study sponsor is Novartis Pharma, Nürnberg (internal study code CAFQ056BDE-EPI-01), and the supervising health authority is the BfArM.

The ethics committee of the University of Rostock approved the study. Patients and caregivers must be informed about the study and data protection issues by written patient information, and written informed consent must be obtained before the patient can be documented.

In 20% of participating centres, on-site monitoring of the centres will be performed with source data verification. Physicians will receive remuneration for participation.

### Data collection

Data will be entered by designated staff at the centres via a secure internet-based data entry form. The system automatically performs completeness and plausibility checks. Quality of life forms filled out by the patient or caregiver will be sent to the contract research organisation (CRO), where they will be entered into the central database by trained staff. Copies of these forms will remain in the patients’ files at the study centres. As data are collected in pseudonymised form in the database (i.e. no patient names, initials, or exact birth dates are included in the central database), the individual centres maintain a patient log list that enables them to identify their patients in the database. Study information will be archived for at least 10 years by the sponsor, CRO, and in the individual centres.

### Adverse events

Information on adverse events (AE) and serious adverse events (SAE) related to the administration of any drug will be collected on standardized forms, regardless of causality, and will include type of event, occurrence, duration, and intensity. The investigator is requested to assess causality and to report actions taken and outcomes. Information on AE and SAE will be processed by the study sponsor, Novartis Pharma, in accordance with established pharmacovigilance rules.

## Discussion

Data are incomplete regarding several aspects of FXS, including epidemiology, clinical and psychosocial characteristics of patients, treatment pathways, and patient-oriented parameters, including quality of life. To the best of our knowledge, the only systematic recording of data takes place in the USA in the context of individual projects, namely by the Fragile X Clinical & Research Consortium (FXCRC), with its FORWARD Registry and Database, and by our Fragile X World Surveys (twice per year, also supported by the U.S. Centers for Disease Control) [14,33-35].

Similar data are needed for Europe, including Germany. The manner and setting of care and specific methods of management of FXS patients throughout the population are not clear. This registry will therefore apply a non-probability approach to collect data on a large sample of patients (convenience sample). While items and variables from the FORWARD Registry and Database will be used to allow comparisons between the registries, a number of items which are specific to the German setting have been added (e.g., setting of care).

For the characterisation of patients and their management, available data from the charts will be used. Further, patients and their caregivers will be requested to fill out established and validated questionnaires to assess the quality of life of patients and caregivers as well as caregiver burden.

Thus, the registry will provide the basis for a comprehensive description of the characteristics and status of FXS patients in Germany in great detail, will allow for gap analyses (comparisons with guidelines), and will likely contribute to the optimisation of management of this vulnerable patient group in the future.

### Trial status

The set-up and testing of the entry form and the database were finalised at the end of 2012. The study is currently open for inclusion, and the first patient was included in March 2013. By the time of this report (14 November 2013), 33 patients have been included. Recruitment is planned until December 2013, and the field (documentation) phase of the study will continue until December 2015. An expansion of the registry to countries other than Germany is also possible. Most study materials are available in English, with the exception of some assessment forms (e.g., IQ testing using validated German instruments).

### Dissemination of information and publications

The study has been registered in ClinicalTrials.gov under NCT01711606, and in the VFA database. The rationale, aims, and design of the study have been previously presented as posters, e.g., at the 2012 meeting of the Deutsche Gesellschaft für Psychiatrie, Psychotherapie und Nervenheilkunde (DGPPN; German Association for Psychiatry, Psychotherapy, and Neurology). A study report will be written upon completion of the study. Further, results will be reported on ClinicalTrials.gov, and the report will be submitted as a peer-reviewed publication.

## Competing interests

MB and HS are full-time employees of Novartis Pharma GmbH, Nürnberg, Germany. The other authors have received honoraria from Novartis Pharma, Nürnberg, for serving on the advisory board of this study, on the Novartis speakers’ bureau, or for research projects related to FXS. They declare no other competing interest.

## Authors’ contributions

All authors made substantial contributions to the design and coordination of the study. FH, DP, HS, and MB wrote the protocol. DP and FH wrote the initial draft of the main text, and all authors approved the final version to be published.

## Pre-publication history

The pre-publication history for this paper can be accessed here:

http://www.biomedcentral.com/1471-244X/13/339/prepub
